# GuavaH: a compendium of host genomic data in HIV biology and disease

**DOI:** 10.1186/1742-4690-11-6

**Published:** 2014-01-15

**Authors:** István Bartha, Paul J McLaren, Angela Ciuffi, Jacques Fellay, Amalio Telenti

**Affiliations:** 1 Institute of Microbiology, University Hospital Lausanne, Lausanne, Switzerland; 2 School of Life Sciences, École Polytechnique Fédérale de Lausanne, Lausanne, Switzerland; 3 Swiss Institute of Bioinformatics, Lausanne, Switzerland

**Keywords:** Genome-wide association studies, Exome analysis, Transcriptome analysis, HIV latency

## Abstract

**Background:**

There is an ever-increasing volume of data on host genes that are modulated during HIV infection, influence disease susceptibility or carry genetic variants that impact HIV infection. We created GuavaH (Genomic Utility for Association and Viral Analyses in HIV, http://www.GuavaH.org), a public resource that supports multipurpose analysis of genome-wide genetic variation and gene expression profile across multiple phenotypes relevant to HIV biology.

**Findings:**

We included original data from 8 genome and transcriptome studies addressing viral and host responses *in* and *ex vivo*. These studies cover phenotypes such as HIV acquisition, plasma viral load, disease progression, viral replication cycle, latency and viral-host genome interaction. This represents genome-wide association data from more than 4,000 individuals, exome sequencing data from 392 individuals, *in vivo* transcriptome microarray data from 127 patients/conditions, and 60 sets of RNA-seq data. Additionally, GuavaH allows visualization of protein variation in ~8,000 individuals from the general population. The publicly available GuavaH framework supports queries on (i) unique single nucleotide polymorphism across different HIV related phenotypes, (ii) gene structure and variation, (iii) *in vivo* gene expression in the setting of human infection (CD4+ T cells), and (iv) *in vitro* gene expression data in models of permissive infection, latency and reactivation.

**Conclusions:**

The complexity of the analysis of host genetic influences on HIV biology and pathogenesis calls for comprehensive motors of research on curated data. The tool developed here allows queries and supports validation of the rapidly growing body of host genomic information pertinent to HIV research.

## Findings

The field of HIV research has adopted genome-wide technologies in order to meet the goal of understanding the complex interplay between host and pathogen. A growing number of approaches allow the interrogation of DNA variation (genome-wide genotyping, exome and whole genome sequencing), RNA variation (transcriptome analyses by gene expression arrays or deep sequencing), as well as large-scale functional screens (gene silencing using siRNA or shRNA, gain of function using gene overexpression). This is complemented with proteome and protein interaction analyses. The objective of these studies is to characterize the behavior of any gene/protein in the context of HIV infection *in vitro* or *in vivo*.

These studies are generally evaluated using strict statistics, which are necessary considering the large number of hypotheses that are simultaneously tested in most genome-wide scans. In addition, many studies require external validation, such as association results in a separate set of infected individuals, or expression results across various biological conditions. Accessing those resources is complex because raw data, or complete sets of analysis statistics are rarely available – or require re-contacting the original sources. Currently, there is a lack of integrated analysis tools by which researchers can easily access well curated data; to reinforce their own observations, for external replication or for generation of novel hypotheses.

**Figure 1:**
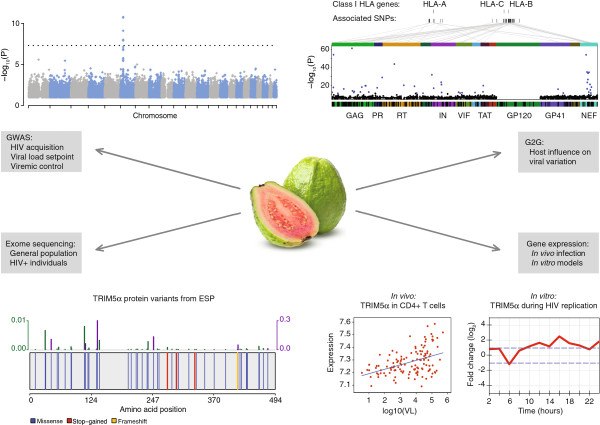
A summary of available data.

Our groups have been involved in the generation and analysis of multiple such large-scale datasets. Thus, we aimed at building a simple platform that would facilitate the comparison of genomic and transcriptomic results across studies, while preserving the scientific interests of researchers and the privacy of study participants. This paper describes the structure of GuavaH (Genomic Utility for Association and Viral Analyses in HIV, http://www.GuavaH.org) and the central issues of interpretation and integration of genome-wide association (GWAS), exome and transcriptome data generated in the context of HIV research (Figure 1).

GuavaH currently provides results from GWAS of HIV disease phenotypes including more than 4,000 individuals. GWAS use large-scale genotyping technology (usually arrays interrogating 500,000 to 1 million single nucleotide polymorphisms, SNPs) complemented with statistical approaches that allow imputation of millions of additional variants that are not directly measured by the assay. The main challenge of GWAS is the stringent statistical threshold for claiming association (usually p < 5 × 10^-8^). The power to identify SNPs associated with a given phenotype depends on the frequency and the effect size of the genetic variant, and on sample size. Thus, large numbers of study participants and meta-analyses across studies are required. GuavaH includes association results on HIV control (set point plasma viral load [1,2] and elite control [3]) and on susceptibility to infection in a cohort of highly exposed seronegative individuals [4]. In addition to these traditional GWAS of clinically related outcomes GuavaH includes data from a recent genome-to-genome analysis of host genetic variants impacting the nucleic acid sequence of the infecting virus [5]. The genome-to-genome approach identifies loci of host-pathogen conflict independently of clinical data. Thus, GuavaH allows the interrogation of any SNP across multiple studies and phenotypes, and facilitates the validation of associations identified in other studies.

**Figure 2:**
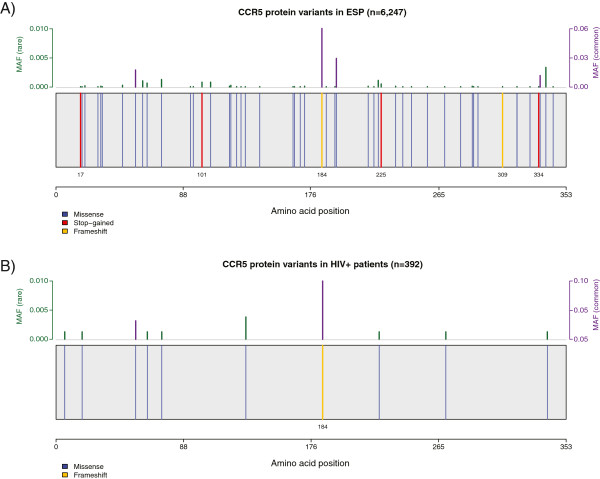
**Exome view of CCR5 in GuavaH.** A protein is depicted in linear form (N to C terminus) with blue vertical lines representing nonsynonymous changes, red vertical lines representing premature stop codons and yellow lines representing frameshifts. The minor allele frequency (MAF) is plotted above for rare variants (MAF < 0.01) in green, and in purple for variants at MAF ≥ 0.01. Panel **A** - the graphic is plotted based on exome sequences from more than 8000 individuals from the general population: there are several rare variants that lead to CCR5 truncation that have not been generally recognized. Panel **B** – Other than CCR5α32 (shown at amino acid position 184) none of these protein truncating variants are present among 392 exomes from HIV-infected individuals.

Large amounts of biological and genomic data are generated by additional emerging technologies. One approach that is transforming genome analysis is the study of human exome variation by high-throughput sequencing. In contrast to genotyping arrays, which only capture relatively common variation, exome sequencing captures all variants present in the coding regions of the genome: common, rare, and private. Each individual harbors about 20,000 unique coding variants, including a number of potentially severe functional variants coding for stop codons and for frameshift insertions or deletions [6]. Analyses are still complex, as there are statistical and functional limitations to the interpretation of rare variants. GuavaH provides gene-level/regional p-values and a graphical representation of nonsynonymous variants, premature stop codons and frameshift variants. We include protein-level sequence variation on a large sample taken from the general population (Analysis of more than 8000 exomes from the Exome Sequencing Project (http://evs.gs.washington.edu/EVS/)), and on 392 HIV infected individuals. Access to exome data in the HIV + sample is restricted due to data protection requirements, but gene-level queries are possible upon request (contact@guavah.org). This detailed level of protein sequence variation information allows for visualization and first-pass estimation of the mutational burden of a given gene (*i.e.* level of conservation or variation) and provides easy access to the genomic location and impact of protein variants in human genes that may be of importance in the HIV life cycle. For example, Figures 1 and 2 present the exome structure of *TRIM5α* and *CCR5*, respectively. For both genes, the report identifies a number of rare premature stop codons.

The GuavaH resource also includes functional transcriptome analyses from *in vivo* and *in vitro* studies. The *in vivo* data were obtained by microarray studies of CD4+ T cells from 127 individuals chronically infected with HIV, and representing the full spectrum of viral load [7]. These data can be contrasted with temporal *in vitro* analysis of the HIV replication cycle in a T cell line (Sup T1), representing 12 data points from HIV infected cells and 12 data points from uninfected cells analysed by sequencing [8]. For example, Figure 1 illustrates the *in vivo* and *in vitro* increase in *TRIM5*α expression during active HIV-1 infection. Given the growing importance of latency research, we also incorporated detailed RNA sequencing data on the dynamic process of entering and maintaining latency in a primary cell model, and on the expression changes in host and viral transcripts upon reactivation with various pharmacological agents and immunological stimuli. GuavaH allows the interrogation of any gene across studies and cellular systems, and facilitates the validation of expression profiles identified in other studies.

GuavaH does not report on some additional large-scale genome-wide data (siRNA, gain-of-function screens) or on HIV-host protein interactions because these data are conveniently available through other open access resources (see [9] and (Table 1)). GuavaH is also linked to other associated resources from our group that allow more detailed and interactive exploration of the genome-to-genome data, of the viral replication cycle dynamics, and on the latency models (Table 1). Expected additions to GuavaH in coming months are proteome and phosphoproteome data, and additional transcriptome datasets from primary cell models of latency.

**Table 1:** Online resources on host genes in HIV biology and disease

**Web site**	**URL**	**Content**
**Associated sites to GuavaH**		
PEACHi	http://peachi.labtelenti.org	Querying of cellular responses to HIV *in vitro* (SupT1 cells)
LITCHi	http://litchi.labtelenti.org	Querying of expression data during HIV latency and upon reactivation in a primary CD4+ T cell model
G2G	http://g2g.labtelenti.org	Interactive HIV-host genome-to-genome map of the HLA class I locus and viral genome variation
**External sites**		
Gene overlapper	http://hivsystemsbiology.org/GeneListOverlapper/	Interactive overlapping of output from genome-wide surveys of host cell genes linked to HIV infection
NCBI HIV-1 Human protein interaction database	http://www.ncbi.nlm.nih.gov/projects/RefSeq/HIVInteractions/	The HIV-1, human protein interaction data are based on literature reports.
Reactome HIV	http://reactome.org	Visualization, interpretation and analysis of pathway knowledge
VirusMINT – Virus molecular interaction database	http://mint.bio.uniroma2.it/virusmint/Welcome.do	Interactions between human and HIV proteins are integrated in the human protein interaction network

Promoting easy access to genome-wide association and functional data fits the goal defined in 2009 by The Global HIV Vaccine Enterprise of understanding the role of host genetics in HIV research*: “New high-throughput genetic approaches have the potential to identify major genetic factors contributing to clinical outcome in HIV-1 infection. Ideally, every human gene that impacts on each mode of HIV transmission and disease outcome should be identified to improve our understanding of the mechanisms of protection*” [10]. GuavaH is a useful tool for visualizing the host genomic effects attributable to a given gene of interest and its potential functional implications in a variety of *in vitro* and *in vivo* settings of HIV infection.

## Availability of supporting data

GuavaH provides access to published datasets and to unpublished data upon discussion with the researchers in charge of the original work. It also allows depositing of new sets of data for public or private querying. Contact: contact@guavah.org

## Competing interests

The authors declare that they have no competing interests. GuavaH is an academic initiative supported with funds from the Swiss National Science Foundation.

## Authors’ contributions

IB developed the web interface, and is responsible for generation of the genome-to-genome data, PM is primarily responsible for generation and curation of genome wide association data, AC is responsible for generation and curation of expression data, JF and AT designed and executed the original studies. All authors contributed to the manuscript and final design of the web interface. All authors read and approved the final manuscript.
